# Effects of multimedia integrated fine arts education on students' learning attitude and learning satisfaction

**DOI:** 10.3389/fpsyg.2022.907468

**Published:** 2022-10-13

**Authors:** Xiaodong Sun, Rong Fu, Guoqing Zhang, Chenin Chen

**Affiliations:** ^1^College of Art, Taiyuan University of Technology, Taiyuan, China; ^2^Fine Arts School, Shanxi Datong University, Datong, China; ^3^Business School, Shanxi Datong University, Datong, China; ^4^Chula Business School, Chulalongkorn University, Bangkok, Thailand

**Keywords:** fine arts education, multimedia instruction, learning attitude, learning satisfaction, technology teaching

## Abstract

The educational reform of the twenty-first century was a successful attempt in which high technology, media, and multimedia computer information work together. The use of instructional media doubled the effect with half the effort in promoting learners' motivation to learn. This was achieved by providing specific and authentic information, changing attitudes, and even being independent of instruction. The rapid development of technology also brings innovations in teaching media. The purpose of this study is to investigate the effects of multimedia integrated fine arts education on students. A quasi-experimental design was adopted in this study. College students from Shanxi Province were selected as the participants of a one-semester experimental study. The research results show significantly positive effects of (1) multimedia integrated fine arts education on learning attitude, (2) multimedia integrated fine arts education on learning satisfaction, and (3). mobile learning on learning satisfaction. The experimental observation revealed that, when fine arts works are exhibited through multimedia, students have closer contact with them. Students gradually feel more involved in art and show interest in art, as well as a desire to explore. Such a process of change will enable students to change their learning attitude into active absorption of professional knowledge of fine arts. As a result, their competence in fine arts can be improved significantly. It is expected that the results will contribute to the fine arts and humanities with practically tested theories as a reference for teachers and future studies in the field.

## Introduction

With the increasing popularity of computers and networks, the use of multimedia as a teaching tool in the learning environment has become a new trend. Online courses in higher education are also becoming more popular (Yakubova et al., 2020). To promote students' concentration and stability in class, the curriculum content for education is developed in a lively and diverse way. Besides, interactive materials are the most important elements of the new curriculum to avoid boring content. Students' participation and their integration in the course content are emphasized to increase the effectiveness of learning and acquisition of knowledge. Moreover, these can enable interaction, reaction, and enthusiasm in the learning process (Stylianou et al., 2019). For this reason, educational reform in the twenty-first century is the flourishing period of an integrated approach that involves high technology, media, and computer information multimedia. The use of instructional media doubled the effect with half the effort in arousing learners' motivation to learn. It also provided specific and authentic information, changed students' attitudes, and enabled an autonomously following instruction. The use of media is not limited to classrooms in schools. With the rapid development of technology, teaching media are constantly renewed, and more and more new technologies are introduced to schools. Tomorrow's world may be impossible to predict, but there is a trail to follow.

If we look at the current situation of fine arts education in China, it can be summarized in four points. 1. The “manual” operation and learning techniques are overemphasized. 2. The educational value of fine arts is in fact stunted. 3. There are “units” but no “curricula”. 4. It is alienated from the “environment” and is “detached”. Fine arts education in national schools put too much emphasis on training and learning. However, the use of media and the practice of skills in teaching activities is getting more widespread, and it is becoming the main focus of education (Bouck et al., 2020). The reform of fine arts teaching leads to the fact that fine arts teaching is no longer just a sheet of drawing paper and several brushes. When considered from a multicultural position, it leads students to learn about themselves, society, and the world. To achieve this teaching goal, fine arts teachers need to demonstrate their professional knowledge and competence, and transform their teaching materials and methods, as well as the instructional media that they use. By doing so, they will be able to meet the diverse and high-tech information. The integration of information technology into teaching is currently one of the most important issues in education. The combination of teaching activities and computer information, networks, and multimedia will be inevitable. The future trend in instructional media is to combine computers with other instructional media to create a student-centered multimedia learning environment (Simonson and Thompson, 1990).

Daniel et al. (2019) predicted that “the network will be the blackboard of the future”, and they added that schools at all levels and learning styles and for different subjects will be driven by computer interaction. In particular, the design of specific multimedia materials for educational courses in research institutions and schools at all levels can greatly enhance students' learning interests. The use of multimedia can also increase the effectiveness of learning. In this respect, in the twenty-first century, the materials and methods for fine arts education should be in line with cultural diversity and integrity. Accordingly, instructional media should be integrated with computer-based instruction, the Internet, multimedia, and hypermedia. When the changing world is considered with its increasing uncertainties, complicated international environment, and rapid changes, it is important for teachers to be able to attract students' attention. Teachers who use traditional dictation, blackboard writing, and playing slides cannot succeed in this in the current environment with diverse sounds and colors. The use of multimedia and hypermedia in instruction has become a trend (Saunders et al., 2018). Pourdavood and McCafferty (2020) pointed out that school education in China does not emphasize fine arts courses. As a result, these courses are conducted ineffectively which cannot arouse students' interest and motivation to learn. The reason behind this dates back to 3–4 decades when fine arts teachers still approached classes as painting teachers, and they completely ignored the cultural significance and connotation of fine arts courses. Therefore, the pedagogical outcomes and achievements of holistic education, integrated education, and multicultural education could not be reached. These curricula were also out of date, and students did not realize the necessity and joy of fine arts learning. To increase the performance and students' willingness and interest in fine arts classes, teachers need to improve teaching materials and adopt appropriate methods. In addition, they should try to use diverse, modern, and multimedia-integrated materials to meet the student's needs and promote their learning interests. Considering the above situations and factors, the effect of multimedia integrated fine arts education on students' learning attitude and satisfaction was investigated in this study. It is expected that the results of this study will contribute to fine arts and humanities education with practically tested theories as a reference for fine arts education teachers and future studies.

## Literature review

### Multimedia integrated fine arts instruction model

Traditional artistic creation media focused on concrete media, such as paper and pencils. The development and increased popularity of software and hardware in the field of information technology has led multimedia to break the traditional structure of instruction. A new form of fine artistic creation has been produced through the integration and application of digital media and traditional media (In'am and Sutrisno, 2021).

1. The data for fine arts classes were transferred into the computer for editing and integration, and presented directly with computer media or converted to other image formats.
2. The data for fine arts classes were transferred into the computer for storage, editing, and integration, and slides were printed as instructional resources.
3. The data for fine arts classes were transferred into the computer for storage and integration, and multimedia software was used to compile the data into interactive instructional software or self-learning software.
4. Online instruction is linked to fine arts education resource websites or the resources were downloaded to integrate with image editing or word processing software.
5. Fine arts lesson data were entered into computers for digital storage and integration and offered on the Internet as shared instructional resources.

In addition, the rich instructional resources on the network can be beneficial to fine arts teaching. The common Internet-based fine arts teaching models are introduced below (Celen, 2020).

(1) Online teaching model

This teaching model directly uses the existing resources by connecting to the Internet.

(2) Offline teaching model

In this teaching model, which is also called airplane mode, network resources are first downloaded to be stored on a hard disk or floppy disk, and then offline instruction is carried out. It is characterized by not being restricted to a network line or transmission bandwidth. In this model, instructors are more mobile, and the re-organization of network platform resources can be organized according to instructors' needs for the instruction.

(3) Thematic teaching model

The instructor first designs a topic according to the learners' level, and the learners use various search methods on the Internet to sort, classify, organize, annotate, or complete reports on the relevant data.

(4) Video-on-demand teaching model

Fine arts teachers can convert various fine arts teaching videos into avi or mpg format, store them on a computer with a larger hard drive, and open them for sharing. The instructional videos can be played anytime and anywhere through networking.

### Effects of multimedia instruction on learning attitude

Ziegler et al. (2021) stated that in a multimedia classroom environment, teachers can add sound effects, texts, or videos to capture the attention of students with different learning styles. In this context, classrooms become attractive, just like theaters. Multimedia can increase the effect of students' learning. 1. Multimedia instruction environment can promote students' learning motivation and attitude. 2. Multimedia instruction environment can promote students' creative thinking. 3. Network multimedia can facilitate students' creative thinking. 4. Multimedia can improve students' flexibility. Experts also pointed out that using interactive computer-based multimedia instruction in education and training can reduce costs by 64% and learning time by 36%. It can also improve students' academic achievement, learning attitude, and comprehension by 11, 28, and 32%, respectively (Taylor and Lee, 2021). In an experiment on perspective drawing, Daniel et al. (2019) discovered that students with computer-assisted instruction showed a more positive learning attitude than those without computer-assisted instruction. Therefore, the following hypothesis is proposed in this study.

**H1**: Multimedia integrated fine arts education has significant positive effects on learning attitude.

### Effects of multimedia instruction on learning satisfaction

Buzhardt et al. (2020) mentioned that compared to other media, multimedia technology has many advantages, such as strong interactivity, a large amount of information transfer, high speed, convenient use, and stimulation of multiple senses. The use of multimedia instruction can be more intuitive, interactive, integrated, controllable, and editable. The use of multimedia in instruction can be a vivid way of presenting the curriculum content, and it can stimulate students' learning motivation and increase their learning satisfaction. Holyfield et al. (2019) believe that the purpose of using multimedia is for communication management. In this respect, it can be stated that multimedia enables adapting the content according to individual differences and expressing the teaching content with optimum media. In addition, multimedia instruction can provide various communication channels for learners to achieve the highest effect and satisfaction. Multimedia that corresponds to human characteristics in terms of multiple senses can provide information in different forms. This can make the transmission and reception of feelings and information more authentic. Moreover, information can be offered according to learners' needs and responses, so that the delivery of information is not limited to only fine arts, but it represents interactive learning and enables individualized learning. Educational experts have confirmed that this can contribute to learning satisfaction (Qahmash, 2018). Accordingly, this study hypothesizes the following.

**H2**: Multimedia integrated fine arts education has remarkably positive effects on learning satisfaction.

### Effects of learning attitude on learning satisfaction

Hord et al. (2020) indicated significant correlations between students' learning attitude and learning satisfaction. According to their study, it can be claimed that the better the learning attitude is, the higher the learning satisfaction will be. Nally et al. (2021) considered the correction of curricula with which learners revealed dissatisfaction, and negative feedback is important for curriculum evaluation and development. In addition, institutions can develop tests in line with the research on satisfaction and remove unsuitable curricula to reduce the likelihood of failure. Learning satisfaction research indicated that improving deficient curricula can enhance learners' positive attitude and guide their development. Ekin et al. (2018) found that the better the learning attitude, the higher the learning satisfaction. They also found that learning attitude is related to learning satisfaction. Learning satisfaction is not simply the indicator of the outcome of the learning activity, but also the main indicator to promote learning motivation and attitude and develop the curricula. Laarhoven et al. (2018) considered that each person has different learning needs and attitude throughout life, and satisfaction is the achievement of these needs or attitudes. Therefore, a prior understanding of the needs and attitudes is necessary for education. Learning satisfaction and learners can achieve the goal expected before learning after participating in a learning activity. In this respect, this study hypothesizes the following.

**H3**: Learning attitude has a significant positive effect on learning satisfaction.

## Methodology

### Measurement of research variable

#### Learning attitude

The dimensions of learning attitude were mentioned by Ok et al. (2021). Accordingly, learning attitude is divided into (1) intrinsic motivation and (2) extrinsic motivation.

#### Learning satisfaction

The concept was clarified by Hunghes (2019) who indicated that learning satisfaction includes two dimensions: (1) teachers' instruction and (2) curricula and environment.

### Participants of the study

In line with the purpose of the study to test the research hypotheses, this study adopts a quasi-experimental design model. College students from Shanxi Province were selected as the participants for the quasi-experimental study. Multimedia integrated fine arts education was implemented in the experimental group, and the control group maintained the traditional teaching model for one semester. SPSS was used for the analysis of the data. The data were analyzed through factor analysis, reliability analysis, regression analysis, and analysis of variance to test hypotheses.

### Data analysis

Analysis of variance was used in this study to discuss the difference between learning attitude and learning satisfaction in the context of multimedia integrated fine arts education. Moreover, regression analysis was implemented to understand the relationships between learning attitude and learning satisfaction.

## Results

### Reliability and validity analysis

Factor analysis was used to extract two factors from learning attitude: “intrinsic motivation” (eigenvalue = 2.487, α = 0.89) and “extrinsic motivation” (eigenvalue = 2.521, α = 0.91). The cumulative covariance explained reached 78.427%.

As for learning satisfaction, two factors were extracted: “teachers' instruction” (eigenvalue = 3.216, α = 0.93) and “curricula and environment” (eigenvalue = 2.831, α = 0.90). The cumulative covariance explained achieved 81.452%.

### Effects of multimedia integrated fine arts education on learning attitude and learning satisfaction

#### Difference analysis of multimedia integrated fine arts education on learning attitude

This study investigates the difference between teaching models and learning attitude using the analysis of variance. Table 1 shows a remarkable difference (*p* = 0.000^*^) between the teaching models and intrinsic motivation. Multimedia integrated fine arts education (4.05) revealed higher intrinsic motivation than the traditional teaching model (3.62). The teaching model also showed a difference (*p* = 0.000^*^) in terms of extrinsic motivation. Multimedia integrated fine arts education (4.11) showed higher extrinsic motivation than the traditional teaching model (3.73). H1 is therefore supported.

**Table 1:** Difference analysis of multimedia integrated fine arts education in terms of learning attitude.

**Variable**		***F***	***P***	**Scheffe post hoc**
multimedia integrated fine arts education	intrinsic motivation	22.324	0.000^*^	multimedia integrated fine arts education (4.05)>traditional teaching model (3.62)
	extrinsic motivation	31.475	0.000^*^	multimedia integrated fine arts education (4.11)>traditional teaching model (3.73)

* p < 0.05.

In the literature on multimedia integrated curriculum, most researchers, such as Daniel et al. (2019) and Ziegler et al. (2021), consider that multimedia integrated instruction can actually enhance the attention of students and help them in terms of increasing their learning motivation. It is considered in this study that fine arts education integrated with multimedia instruction can stimulate students' learning and enhance the learning freshness. The researcher observed students' behavior during the research and discovered that some students actively watched multimedia instructional videos and made discussions after class, which showed that this approach enhanced their learning motivation.

#### Difference analysis of multimedia integrated fine arts education in learning satisfaction

The difference between teaching models and satisfaction with teachers' instruction was discussed according to the results of the analysis of variance. Table 2 shows notable differences (*p* = 0.000^*^) between teaching models and learning satisfaction. Multimedia integrated fine arts education (3.88) reveals higher results in terms of teachers' instruction with multimedia than using the traditional teaching model (3.56). The teaching model shows significant differences (*p* = 0.000^*^) in learning satisfaction with curricula and environment. Multimedia integrated fine arts education (4.37) showed a higher value for curriculum and environment than the traditional teaching model (3.84).

**Table 2:** Difference analysis of multimedia integrated fine arts education in learning satisfaction.

**Variable**		***F***	***P***	**Scheffe post hoc**
multimedia integrated fine arts education	teachers' instruction	33.187	0.000^*^	multimedia integrated fine arts education (3.88)>traditional teaching model (3.56)
	curricula and environment	46.787	0.000^*^	multimedia integrated fine arts education (4.37)>traditional teaching model (3.84)

* p < 0.05.

Instructors' use of rich teaching content contributes to increasing students' learning. Teachers' attitude and teaching skills are important for students. Even when teachers use different teaching styles which do not appeal to students, it can reduce students' learning satisfaction to a great extent.

### Correlation analysis of learning attitude and learning satisfaction

#### Correlation analysis of learning attitude and teachers' instruction

The results of the analysis conducted to test H3 are presented in Table 3. According to the results, it can be stated that learning attitude has significant effects on teachers' instruction (*p* = 0.000^***^). Moreover, it has remarkably positive effects on intrinsic motivation (β =2.312^**^) and extrinsic motivation (β = 2.221^**^).

**Table 3:** Analysis of learning attitude to learning satisfaction.

**Dependent variable→**	**Learning satisfaction**			
**Independent variable↓**	**Teachers' instruction**		**Curricula and environment**	
**Learning attitude**	**β**	***P***	**β**	***P***
Intrinsic motivation	2.312^**^	0.000	2.546^**^	0.000
Extrinsic motivation	2.221^**^	0.000	2.465^**^	0.000
*F*	35.287		43.625	
Significance	0.000^***^		0.000^***^	
*R* ^2^	0.327		0.417	
Adjusted *R*^2^	0.306		0.394	

*p < 0.05,

** p < 0.01.

Self-organized in this study.

#### Correlation analysis of learning attitude and curricula and environment

The results of the analysis conducted to test H4 are presented in Table 3. The results showed that learning attitude has remarkable effects on the curriculum and environment (*p* = 0.000^***^). Similarly, it has significant positive effects on intrinsic motivation (β = 2.546^**^) and extrinsic motivation (β = 2.465^**^). Therefore, H4 was supported. The results of the hypothesis test are shown in Table 4.

**Table 4:** Hypothesis test.

**Research hypothesis**	**Correlation**	**Empirical result**	***P***	**Result**
H1	Intrinsic motivation	Multimedia integrated fine arts education (4.05)> traditional teaching model (3.62)	0.000^*^	Supported
	Extrinsic motivation	Multimedia integrated fine arts education (4.11)> traditional teaching model (3.73)	0.000^*^	
H2	Teachers' instruction	Multimedia integrated fine arts education (3.88)> traditional teaching model (3.56)	0.000^*^	Supported
	Curricula and environment	Multimedia integrated fine arts education (4.37)> traditional teaching model (3.84)	0.000^*^	
H3	Teachers' instruction	Intrinsic motivation (β = 2.312^**^), Extrinsic motivation (*β =* 2.221^**^)	0.000^***^	Supported
	Curricula and environment	Intrinsic motivation (*β =* 2.546^**^), Extrinsic motivation (*β =* 2.465^**^)	0.000^***^	

* p < 0.05,

** p < 0.01,

*** p < 0.001.

Self-organized in this study.

## Discussion

In fine arts education, proper learning situations should be created to provide diverse multimedia teaching methods and properly implement the teaching elements of “situation”, “material”, and “method”. When teachers integrate fine arts education into multimedia teaching activities, students can be interested and attracted to the learning activity. Nevertheless, teachers should consciously consider these three elements, as well as students' experience, background, and previous knowledge, during continuous teaching activities to maintain learning interest. Various challenging activities and games can add variety to the lessons to meet students' learning needs and interests and achieve teaching goals. The correlation between learning motivation and learning effect should be emphasized when designing lessons for actual practice. The learning activities should take into account students' individual differences. Various successful cases should be offered in the practice of the whole teaching design to encourage students to develop their self-expectation according to their personal abilities. In addition, the possibilities of lack of learning or having negative attitudes toward learning caused by learning frustration can also be prevented. A good teaching design does not necessarily adopt a single teaching method. Using a variety of multimedia teaching methods that cater to students with different learning experiences and backgrounds allows students to choose the most appropriate and effective learning style for themselves.

Multimedia teaching is diverse, simple, and convenient, but it cannot replace the interaction between teacher and student as in traditional teaching. Students often encounter difficulties and ambiguities in the learning process that require teachers' immediate responses. In this respect, when using multimedia teaching, teachers should not just consider the content as rich and cut short or ignore students' feedback. Teachers should point out and emphasize students' feedback in fine arts education, apply the function of multimedia teaching well, utilize audiovisual features, use alternative interactivity with students, and supplement traditional teaching to achieve positive effects on teaching.

The above-mentioned results revealed that effective and interesting teaching strategies can lead students to be satisfied with their learning, have a pleasant learning experience, and achieve the objectives autonomously. These feelings result from the comprehensive applications of material development, purposive design of learning activity, development of instructional media, and improvement of learning evaluation methods. As a result of these approaches, the courses will present higher value and tension, and will also allow learners to enjoy learning.

## Conclusion

The results of the experimental research showed that students did not have any negative perceptions with regard to aesthetics, history, and fine arts. Students showed no interest in fine arts and aesthetics, which may be because of the strangeness, rarity, unfamiliarity, and lack of experience. These may lead students to fear, distancing, and exclusion. When fine artworks are exhibited through computer multimedia, most students in this study had the opportunity to engage with the fine artworks. Most of the students had close contact with the fine artworks, which helped them to eliminate the strangeness and exclusion with a brief introduction by teachers. As a result, they showed interest, novelty, and curiosity to explore. Such a change enables students to acquire expertise and competence in fine arts, which leads them to take part in fine arts more actively. The research results conform to the studies of Qahmash (2018), Daniel et al. (2019), Holyfield et al. (2019), and Taylor and Lee (2021).

Instructional principles are the theoretical basis of instructional methods. Flexibly applying various innovative instructional methods and improving teaching activity can lead to excellent teaching effectiveness (Hunghes, 2019). Teaching activities should focus on students' learning, and students should be placed at the center of the learning process. Teachers are both instructors and counselors. Therefore, they should promote and facilitate students' learning and encourage students to observe, experience, do, and think (Celen, 2020). The expansion of multimedia instruction means the expansion of students' learning. In addition, it contributes to the transmission technology as well. Similarly, the use of multimedia improves the effects of visual images, which meets people's visual needs. At the same time, computers provide sound effects and dynamic 3D images to attract people. Combining the brilliant performance of computer multimedia with fine arts education, especially with the visual fine arts, plays the role of an interface between fine arts teachers and students. Particularly, the rich multimedia types enable fine arts teachers to choose different suitable media according to the characteristics and features of the curriculum content. The combination of 2–3 types of multimedia can enrich the internal content and external presentation of curricula, and present the richness and excellence of curricula. Also, the specialty, seriousness, and responsibility of teachers can make students respect curricula and teachers. As a result, their learning attitude and learning satisfaction is improved. In addition, the use of 2–3 types of multimedia can expand the transmission interface between fine arts teachers and students, as well as between instruction and students. This provides students with more opportunities to absorb information and improve the effectiveness of learning. Compared to learning fatigue, visual fatigue, auditory fatigue, and psychological freshness fatigue caused by a single instructional medium, the use of 2–3 types of multimedia can improve learning attitude and learning satisfaction.

## Data availability statement

The original contributions presented in the study are included in the article/supplementary material, further inquiries can be directed to the corresponding author/s.

## Ethics statement

The present study was conducted in accordance with the recommendations of the Ethics Committee of the Shanxi Datong University, with written informed consent being obtained from all the participants. All the participants were asked to read and approve the ethical consent form before participating in the present study. The participants were also asked to follow the guidelines in the form in the research. The research protocol was approved by the ethical committee of the Shanxi Datong University.

## Author contributions

XS performed the initial analyses and wrote the manuscript. XS, RF, GZ, and CC assisted in the data collection and data analysis. All authors revised and approved the submitted version of the manuscript.

## Funding

The authors appreciate financial support for the research and publication of this fine article (2021YY167) from Shanxi Provincial Office of Philosophy and Social Science Planning, Shanxi Province, China.

## Conflict of interest

The authors declare that the research was conducted in the absence of any commercial or financial relationships that could be construed as a potential conflict of interest.

## Publisher's note

All claims expressed in this article are solely those of the authors and do not necessarily represent those of their affiliated organizations, or those of the publisher, the editors and the reviewers. Any product that may be evaluated in this article, or claim that may be made by its manufacturer, is not guaranteed or endorsed by the publisher.
